# Stellate ganglion block: what else is necessary to include in the treatment of subarachnoid hemorrhage patients?

**DOI:** 10.1186/s41016-024-00374-3

**Published:** 2024-08-01

**Authors:** Leonardo C. Welling, Nicollas Nunes Rabelo, Mateus Gonçalves de Sena Barbosa, Beatriz Rodrigues Messias, Carolina Guimarães Pinto, Eberval Gadelha Figueiredo

**Affiliations:** 1 Department of Neurosurgery, State University of Ponta Grossa, Ponta Grossa, PR Brazil; 2 Division of Neurosurgery, School of Medicine, University of São Paulo (FMUSP)/Hospital das Clínicas, Dr. Enéas de Carvalho Aguiar Street, 255, São Paulo, SP 05403-010 Brazil; 3 Department of Neurosurgery, Atenas Medical School, Passos, MG Brazil; 4 Department of Neurosurgery, Albert Einstein Medical School, São Paulo, SP Brazil

**Keywords:** Cerebral vasospasm, Subarachnoid hemorrhage, Stellate ganglion block

## Abstract

Cerebral vasospasm is determined as a temporary narrowing of cerebral arteries a few days after an aneurysmal subarachnoid hemorrhage. The onset of this vascular event usually evolves with new neurological deficits or progression of ischemic areas. The success of interventions to treat or revert this condition is not satisfying. In addition to cerebral vasospasm, early brain injury plays an important role as a contributor to subarachnoid hemorrhage’s mortality. In this sense, stellate ganglion block appears as an alternative to reduce sympathetic system’s activation, one of the main pathophysiological mechanisms involved in brain injury. Over the past few years, there is growing evidence that stellate ganglion block can contribute to decline patient morbidity from subarachnoid hemorrhage. Is it time to include this procedure as a standard treatment after aneurysm rupture?

## Main text

Cerebral vasospasm (CV) is a major cause of neurological morbidity in patients who survive aneurysmal subarachnoid hemorrhage (SAH). Also, mortality increases by up to three times in the first 2 weeks. Numerous treatments for this condition have been used, including triple-H therapy (hypervolemia, hypertension, and hemodilution), interventional neuroradiological procedures such as transluminal angioplasty, administration of calcium channel antagonists (nimodipine), HMG-CoA reductase inhibitors (statins), and endothelin-1 antagonists (clazosentan). These interventions’ success rate is very limited, and up to 38% of patients develop neurological sequelae or progress to death [1–3].

In addition to CV, recent evidence shows that, in the first 72 h, there is direct damage to brain tissue with increased intracranial pressure and decreased cerebral blood flow. Several inflammatory mediators are involved. Some authors consider that early brain injury (EBI) has a more important effect than vasospasm on mortality. Regardless of the overlapping of pathophysiological mechanisms, the sympathetic system’s activation is involved in EBI and CV [4, 5].

The stellate ganglion (SG) is the main representative of the sympathetic system in the craniocervical region. The SG is composed of the lower cervical ganglion and the upper thoracic ganglion. Stellate ganglion block (SGB) is used to block sympathetic innervation of the head and neck temporarily, inducing peripheral vasodilation in these regions [6]. In 2003, Treggiari et al. [6], based on the concepts of activation of the sympathetic system after SAH, carried out a study in which the sympathetic cervical block was used to treat CV. Although there was no improvement in the large vessel caliber, there were indirect signs of better cerebral perfusion, filling small parenchymal defects compared to cerebral angiography before SGB [6].

About 10 years later, the underlying molecular mechanisms of SGB began to be explored. The expression of two factors involved in CV, endothelin-1 (ET-1) and calcitonin gene-related peptide (CGRP), was evaluated in animal models [7]. ET-1 is the most potent known endogenous vasoconstrictor, expressed during ischemic insult. It binds to specific receptors on smooth muscle cells and causes constriction of blood vessels and proliferation of endothelial cells [8]. Increased levels of ET-1 are found in plasma and cerebrospinal fluid (CSF) in patients with SAH, suggesting that it may directly contribute to vasospasm [9].

On another side, CGRP is an intrinsic vasodilator peptide, highly potent, released by sensory nerves and found in the perivascular nerve fibers of intracranial arteries. In different models, the CGRP peptide has played a protective role against pro-hypertensive systems, including the sympathetic nervous system [10]. This peptide was also elevated in human patients with proven vasoconstriction at the time of analysis [11]. However, it is reduced in patients who have succumbed to the inflammatory cascade, suggesting that is possibly released in response to vasoconstriction caused by SAH but is depleted after this process is completed. When depleted, it triggers the development of delayed spasm [12, 13]. Thus, CGRP proves to be highly relevant to the mechanism of vasospasm in SAH.

According to Hu et al. [6], SGB significantly decreased ET-1 expression while increasing CGRP expression, possibly resulting in a molecular cascade that favors vasodilation, as evidenced by measurements of cross-sectional area, perimeter, and diameter of the basilar and middle cerebral arteries, which increased significantly after SGB in the presence of SAH. Thus, it is suggested that SGB can relieve vasospasm by regulating plasma concentrations of ET-1 and CGRP. The physiological mechanism by which SGB regulates these factors is not yet clear and requires further studies in the future. Besides, through immunohistochemistry, it was observed that anti-apoptotic pathways were activated by suppressing the Bax protein and activating Bcl-2 in the hippocampus of rats with SAH [6]. These findings indicate that SGB also exhibits a neuroprotective effect on hippocampal neurons.

Recently, Zhang et al. [14] conducted a randomized clinical trial with 102 patients who had suffered SAH and underwent craniotomy for treatment of intracranial aneurysm. One group underwent SGB, while the other received standard treatment (nSGB). They evaluated EBI biomarkers’ levels (IL-1β, IL-6, TNF-α, ET-1, NPY, NSE, and S100β) and hemodynamic parameters, including the mean cerebral blood flow of the middle cerebral artery (Vm-MCA) and the basilar artery (Vm-BA), using transcranial Doppler. The authors observed that the increase of inflammatory markers levels was lower in the SGB group, including IL-1β, IL-6, and TNF-α. Vm-MCA and VM-BA increased in both groups from 1 to 7 days post-surgery compared to baseline; nonetheless, this increment was lower in the SGB group. The changes of vascular physiological markers (ET-1 and NPY) and brain injury markers (NSE and S100 β) followed similar patterns. The proportion of patients with favorable clinical outcomes, considering parameters such as the Glasgow Coma Scale (GCS), motor, and cognitive neurological deficits, was 54% in the SGB group compared to 32.6% in the standard treatment group. These positive results persisted after 6 months, indicating a favorable prognosis in the SGB group [14].

Some studies show that SGB can improve the prognosis of patients with SAH, by preventing the inflammatory response during EBI, reducing endothelial dysfunction, and possibly facilitating the prevention of CV [4, 6, 14]. Furthermore, it stands out as a minimally invasive procedure, already used for other conditions related to pain, traumatic brain injury, and cerebral hemorrhage, demonstrating safety when performed in these situations [14]. SGB does not induce a systemic response when performed correctly. As a regional anesthetic block, guided or not by simultaneous ultrasound, it can be performed by a neurosurgeon or anesthetist, making it a simple and economically accessible procedure [15]. This approach is noteworthy, especially when compared to more invasive surgical methods and is feasible in hospital centers already prepared for treatment of SAH.

In a recent animal study published in 2023 [16], researchers aimed to establish the methodology of ultrasound-guided SGB in rats. Despite the study’s limitations, including a small sample size and challenges in comparing certain complications such as hemothorax and pneumothorax, the authors concluded that ultrasound guidance notably decreased operative duration and the incidence of complications (brachial plexus block, vagal block, respiratory depression, and mortality).

Kirkpatrick et al. [17] classified SGB complications into local and systemic categories, with local issues encompassing intra-procedural bleeding and hematoma formation. A meta-analysis [18] examining SGB-related complications identified hoarseness and light-headedness as the most prevalent systemic adverse effects, with short-term and persistent cough also reported. The main contraindication to the procedure was use of anticoagulants. In a retrospective study by Aleanakian [19] involving 809 ultrasound-guided SGB cases, complications occurred at a rate of 13.2%, with three cases deemed potentially life-threatening. Despite existing literature affirming the safety of SGB, particularly when conducted under ultrasound guidance, most complication reports stem from case studies or retrospective analyses, potentially impeding the broader adoption of SGB.

Deng et. al. [20] comprehensively assessed the therapeutic potential of SGB across a diverse spectrum of conditions, encompassing pain management, immunological disorders, and psychological ailments. Despite the minimally invasive and safe nature of ultrasound-guided SGB, its widespread acknowledgment among specialists remains limited, and consensus regarding its routine application in SAH treatment is lacking.

Given the promising but still inconclusive results surrounding the efficacy of SGB as a standard treatment of SAH, there is a pressing need for further research, particularly through large-scale randomized clinical trials, with longer follow-up periods, and cost-effectiveness studies. It is important to establish the standard procedure of SGB, optimal timing, and long-term effects. These studies should also include the analysis of inflammatory markers and vasoconstriction factors to further solidify the efficacy and safety of the proposed new method at a higher level of evidence.

To comprehensively evaluate SGB’s potential, future studies must not only focus on biochemical markers but also incorporate detailed clinical analyses and prognostic factors, including neurological deficits, complications, and mortality. Such multi-faceted evaluations will provide a more holistic understanding of SGB’s therapeutic value, guiding its optimal integration into clinical practice and ensuring that patient outcomes are maximized in both efficacy and cost-efficiency.

Jing et al. [21] recently published a study protocol for an ongoing randomized clinical trial designed to evaluate the effectiveness and safety of early SGB, administered within 48 h of aneurysmal SAH as a preventive treatment for CV and delayed cerebral ischemia. In this study, 228 patients will be randomized into two groups: one group will receive an additional early SGB before surgical management, while the other group will undergo standard treatment. The primary outcome measure is the incidence of symptomatic CV. Secondary outcomes include the modified Rankin Scale score, incidence of complications, and all-cause mortality. The findings could significantly impact clinical practice by offering a novel approach to improving patient outcomes after SAH.

We already have pathophysiological explanations, whether in CV or in EBI, and laboratory markers that corroborate the best clinical outcomes observed. Is it little to include SGB in the standard treatment routine of patients with SAH?

## Data Availability

Not applicable.

## Abbreviations

CGRP: Calcitonin gene-related peptide
CV: Cerebral vasospasm
CSF: Cerebrospinal fluid
EBI: Early brain injury
ET-1: Endothelin-1
GCS: Glasgow Coma Scale
SG: Stellate ganglion
SGB: Stellate ganglion block
SAH: Subarachnoid hemorrhage
Vm-BA: Mean cerebral blood flow of basilar artery
Vm-MCA: Mean cerebral blood flow of middle cerebral artery
